# Perceived Barriers in Delivering Optimal Healthcare Services in a Dedicated COVID-19 Hospital: Perspectives of Health-Service Providers

**DOI:** 10.7759/cureus.30188

**Published:** 2022-10-11

**Authors:** Nipa Singh, Ipsa Mohapatra, Subhasish Singh, Varsha Srivastava, Krishna Mishra

**Affiliations:** 1 Department of Microbiology, Kalinga Institute of Medical Sciences, Bhubaneswar, IND; 2 Department of Community Medicine, Kalinga Institute of Medical Sciences, Bhubaneswar, IND; 3 Department of Cardiology, Maharaja Krushna Chandra Gajapati Medical College, Berhampur, IND

**Keywords:** covid-19 hospital, health-service providers, covid-19, healthcare, optimal, barriers

## Abstract

Background

The coronavirus disease 2019 (COVID-19) pandemic challenged the healthcare infrastructure, with health-service providers (HSPs) offering unconditional and unprejudiced service to admitted patients. During the first wave, due to the novelty of the disease and the lack of clarity regarding its transmission in the initial phases of the evolution of the disease, the predominant fear was of contracting the disease while caring for patients. With the prevailing uncertainty in knowledge and management, this study was planned to identify the barriers to delivering optimal healthcare to COVID-19 patients.

Methodology

A cross-sectional study was conducted among HSPs working in the first phase of a dedicated 500-bed government COVID-19 hospital at Kalinga Institute of Medical Sciences using an online questionnaire with the following five aspects: workplace guidelines and support, protective equipment, access to information regarding updates on the epidemic, overall self-reported stress and workplace stress about self-infection with COVID-19 and family being infected, and demographics. All HSPs aged 18 years or above, who were working either on a full- or a part-time basis, were able to understand the English language, and who were working in the COVID-19 hospital and gave digital informed consent (via Google Forms) were included in the study. All data were collected, coded, tabulated, and analyzed using Google Forms in an Excel format and Epi Info software version 7.2.5.0.

Results

Of the 144 respondents contacted, 132 completed the survey, with a participation rate of 91.67%. About 52.27% of respondents were aged 21-30 years, 68.18% were females, and 56.06% were nurses. Challenges faced were “working in a new context” (40.91%), “the uncertainty and fear of being infected and infecting others”(31.06%), and “exhausted by the workload and protective gear” (18.94%). Moreover, 64.12% were aware of a workplace policy. Only 0.75% felt that their workload needed to be reduced; 2.27% felt the need for a penalty policy for hiding travel history, lack of quarantine compliance, avoiding the accumulation of face masks, and price inflation of face masks. The overall self-reported stress level was significantly associated with a lack of awareness of workplace policies and the fear of getting infected. Furthermore, 93.94% reported that they had an adequate supply of personal protective equipment. As high as 81.06% of the HSPs were “worried about being infected from COVID-19 during work,” and 94.69% were “worried about their family being infected from COVID-19 due to their working in COVID-19 hospitals.”

Conclusions

HSPs’ perception of barriers in providing healthcare gave an insight into the problems being faced and helped improve the quality of services. The study highlighted the need of increasing awareness regarding the existing workplace policies among HSPs to promote preparedness during crisis management.

## Introduction

The coronavirus disease 2019 (COVID-19) pandemic challenged the healthcare infrastructure of even the most developed nations. Health-service providers (HSPs) who were at the forefront of managing affected patients worked under extremely challenging conditions [1]. Being a novel disease without a definite cure, the job of HSPs was extremely difficult. Some of the challenges included working for long hours wearing personal protective equipment (PPE), lack of concrete guidelines to manage the disease, fear of exposure to the virus, and stigma attached to workers catering to COVID-19 patients. HSPs not only faced difficulties in handling patients but also faced the daunting task of keeping their own worries and emotional stress at bay [2]. During the first wave, with the prevailing uncertainty in knowledge and management, this study was undertaken to assess the concerns and barriers faced by these workers while providing healthcare to COVID-19 patients. Keeping this in mind, this study intended to identify the barriers to delivering optimal health care to COVID-19 patients, the experience of HSPs in handling stigma and stress during their duty, and their views toward workplace supportive policy and PPE supply.

This article was previously presented as an oral paper presentation at the 49th Indian Association of Preventive & Social Medicine (IAPSM) National Conference on March 4, 2022.

## Materials and methods

Using a cross-sectional study design, this study was planned among HSPs working in a 500-bed dedicated COVID-19 hospital during the first wave of the COVID-19 pandemic. The study included all HSPs aged 18 years or above and working either on a full- or part-time basis. HSPs who were able to understand English and gave digital informed consent (via Google Forms) were included in the study. HSPs who could not be contacted and did not have an electronic device with which they could access the internet for completing the questionnaire were excluded from the study. The study tool was a researcher-made Google questionnaire with five aspects, namely, workplace guidelines and support, availability of PPE, access to information for updates on the epidemic, overall self-reported stress, and workplace stress (about self-infection with COVID-19 and family being infected). The questionnaire was made after a thorough literature search [1-3]. HSPs were defined as health workers who delivered direct services to patients, whether personal or non-personal [4].

Respondents were recruited through a convenience sampling technique. The online self-administered questionnaire was promoted by distributing the link to the survey through email and social networking sites to reach the target population. Respondents were encouraged to forward the survey links to others who were on duty with them. The survey was available as a Google Form. An information page was included at the beginning of the survey and respondents’ consent was obtained before they started the survey. All participation was voluntary and anonymous. Respondents who chose to withdraw from the study at any point were allowed to do so. The collected data were stored in Google Drive and password-protected. Thus, only the investigators had access to the account for using the data for analysis. All data were collected, coded, tabulated, and analyzed using Google Forms, Excel format, and Epi Info software (version 7.2.5.0). The study was approved by the Institutional Ethics Committee of Kalinga Institute of Medical Sciences (KIIT/KIMS/IEC/387/2020 dated September 9, 2020).

## Results

Of the 144 HSPs working in the first phase of the pandemic in the 500-bed dedicated COVID-19 hospital contacted, 132 completed the survey, with a participation rate of 91.67%. The mean age of the study participants was 31.58 ± 8.13 years, with a range of 21 years to 51 years, and the majority were females (Table 1).

**Table 1:** Characteristics of the study participants (N = 132). *: Other categories of HSPs, such as pharmacists, lab technicians, etc., were not working in the COVID-19 hospital during the first wave and are not included as participants. HSP: health-service provider; COVID-19: coronavirus disease 2019

	Frequency (in number)	Frequency (in percentage)
Age group (in years)		
18–20 years	0	0.00
21–30 years	69	52.27
31–40 years	36	27.27
41–50 years	26	19.70
51–60 years	1	0.76
Gender		
Male	42	31.82
Female	90	68.18
Type of HSPs*		
Medical doctor	57	43.18
Nurse	74	56.06
Radiology/X-ray technician	1	0.76
Provided direct care to a confirmed COVID-19 patient		
Yes	88	66.67
No	44	33.33

**Figure 1:**
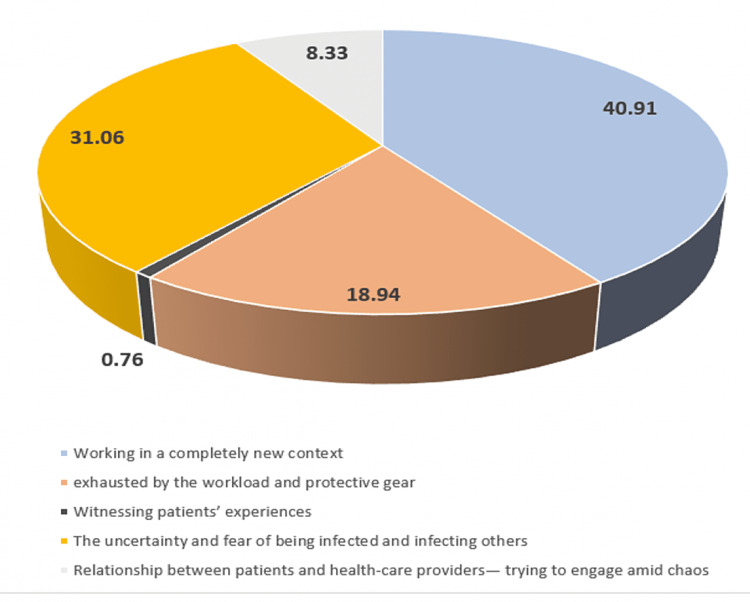
Challenges faced while working in a COVID-19 hospital (N = 132). COVID-19: coronavirus disease 2019

The respondents were asked to choose the most common challenge they experienced while working in the COVID-19 hospital from the choices listed in the questionnaire. The responses are presented in Figure 1.

**Figure 2:**
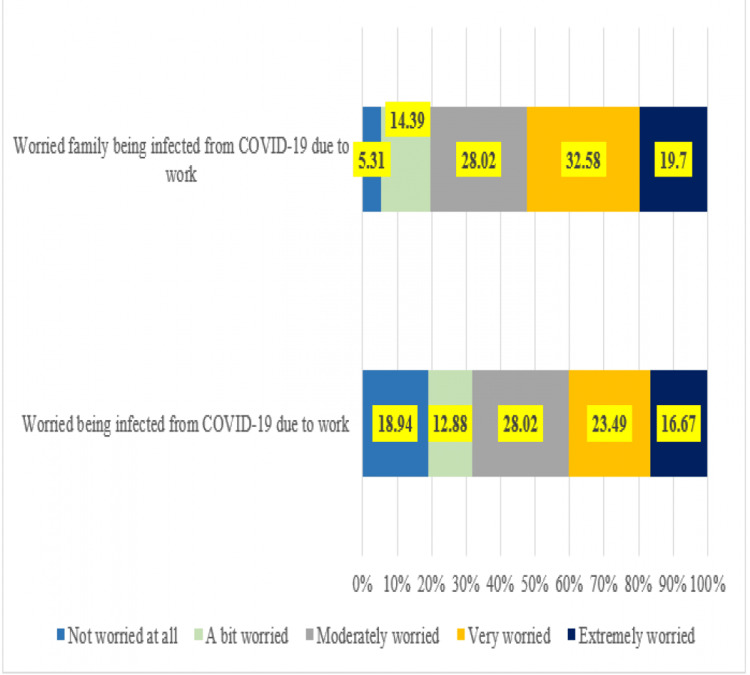
Reasons cited for stress while working in a COVID-19 hospital (N = 132). COVID-19: coronavirus disease 2019

Around 77.10% of the respondents self-reported being stressed in the past seven days (Figure 2). Around 64% were aware of a workplace policy. HSPs were asked to share their views by responding to a set of statements and were asked to tick yes to all that applied to their workplace. The statements were regarding “experience with the workplace policy” and “what needs to be done to strengthen it further.” Only 0.75% felt that the workload needed to be reduced; 2.27% felt the need for a penalty policy for hiding travel history, non-compliance to quarantine, avoiding the accumulation of face masks, and price inflation of face masks. Moreover, 19.69% felt the need for promotion of stress management in the workplace, e.g., relaxation exercises, breathing, music, and workplace design. Overall, 25.76% felt that they were not provided timely information and knowledge about COVID-19. The overall self-reported stress level was significantly associated with “a lack of awareness of workplace policy” (p = 0.02) in place and “the fear of getting infected” (p < 0.001). Additionally, 93.94% reported that they had an adequate supply of PPE.

## Discussion

In this study conducted among HSPs to find their experiences and concerns while delivering healthcare to patients suffering from COVID-19 during the first wave of the pandemic, the participation rate was 91.67%, with 132 of 144 contacted responding to the survey. Similar studies done in different parts of the world which involved a large number of participants and studies that involved a small group of participants reported a high participation rate [5,6]. This gives us an insight that the question of problems and hurdles faced by HSPs is a matter of concern globally. The participation rate in this study is higher in comparison to a study done by Kayama et al. where the participation rate was 75.6% [7]. In this study, females (68.18%) outnumbered males (31.82%) and the greater number of female HSPs in such surveys is substantiated by studies in Saudi Arabia and Japan where female respondents were 70% and 71.5%, respectively [5,7]. This may be because nurses comprise a sizeable proportion of our health workforce. The mean age of participants in this study was 31.58 ± 8.13 years, with a range of 21-51 years. In a study by Almagrabhi et al., the participants were in the age range of 26-34 years. In another study in Pakistan, the maximum proportion (74.9%) of participants were less than 30 years [5,8]. The number of nurses (56.06%) was higher than that of doctors (41.8%) in this study. Studies to evaluate the concerns of healthcare workers have been conducted in different parts of the world and the numbers have been similar [7,9].

Healthcare professionals as a workforce are trained to meet any medical emergency firmly and with tenacity. However, the uncertainty of the pandemic regarding the novel disease and strategies to deal with it overwhelmed the healthcare system. Consequentially, the primary concern (40.91%) that emerged in the study was “working in a completely new context,” followed by other issues such as “being exhausted by workload and protective gear” (31.06%), and “uncertainty of being infected and infecting others” (18.94%). In a review on healthcare worker wellness, respondents in a study in Hubei described working in a new context as a primary challenge along with the fear of infection and exhaustion as points of concern [10]. In the current pandemic, the fear of being infected loomed large but a significant proportion was also concerned about being a source of infection to others [11]. In this study, 31.06% of respondents experienced stress due to fear of being infected and infecting others. Healthcare providers are obligated to work in healthcare institutions, but, in a developing country like India where there is a shortage of personnel even in non-COVID-19 situations, the increased workload during the pandemic put tremendous physical pressure leading to mental stress [11]. In the present study, 18.94% experienced workload and they listed this as the third most common challenge. Overall, 77.1% reported being stressed in the last seven days. Stress was assessed in terms of two parameters: being worried that the family would be infected by COVID-19 due to work, and second being that they (i.e., the HSP) would be infected by COVID-19 due to work. Around 33% of them were “very worried” regarding their family’s safety and 28.02% were “moderately” worried. Similar (12.5%) findings were seen among frontline healthcare workers who experienced stress while performing their duties due to the lack of knowledge regarding the effects of COVID-19 [10]. The concerns of healthcare providers with respect to family issues could be mitigated to a certain extent by crisis counseling, incentives, and financial support [5]. The administration needs to ensure the care of healthcare workers’ family members to enhance workforce confidence and availability [12].

In this study, a significant proportion of participants (25.76% and 19.69%, respectively) were of the opinion that timely information regarding COVID-19 would be helpful and that stress management at the workplace would significantly alleviate the problems of healthcare providers. Provision or inadequacy of PPE was not a matter of concern in the current study as 93.94% said that they had an adequate supply of PPE. In a study done in Saudi Arabia, the healthcare providers provided suggestions regarding the best methods to manage stress during the pandemic, and a majority (45.2%) opined that providing all kinds of PPE would be helpful. Around 37% suggested providing incentives and 14% wanted the prevention of disinformation via social media [5].

This study would have gained more relevance if it would have been possible to include more HSPs from different COVID-19 hospitals. The barriers and challenges identified in the current study may not be representative of the prevailing situation at all levels of healthcare providing COVID-19 services. This study being conducted in a COVID-19 hospital attached to a tertiary care center would have been a reason for the difference in the quality of care and access to specialized services.

## Conclusions

The perception of HSPs to barriers in providing healthcare gave an insight into problems being faced and helped improve the quality of services. The barriers that were identified in this study were “working in a completely new context” and “being exhausted by workload and use of protective gear.” These barriers were addressed by recruiting more HSPs in COVID-19 hospitals and decreasing the working hours in each shift. The study highlighted the need to increase awareness about the existing workplace policies among HSPs to promote preparedness during crisis management. The study results were shared with government officials, and the state of Odisha was later awarded as one of the best performing states in the management of COVID-19, and the institute where the study was conducted was adjudged as the best hospital giving COVID-19 care at the national level.
